# Underwater Monitoring Networks Based on Cable-Structured Triboelectric Nanogenerators

**DOI:** 10.34133/2022/9809406

**Published:** 2022-02-03

**Authors:** Yihan Zhang, Yingying Li, Renwei Cheng, Shen Shen, Jia Yi, Xiao Peng, Chuan Ning, Kai Dong, Zhong Lin Wang

**Affiliations:** ^1^CAS Center for Excellence in Nanoscience Beijing Key Laboratory of Micro-Nano Energy and Sensor, Beijing Institute of Nanoenergy and Nanosystems, Chinese Academy of Sciences, Beijing 101400, China; ^2^School of Nanoscience and Technology, University of Chinese Academy of Sciences, Beijing 100049, China; ^3^CUSTech Institute of Technology, Wenzhou, Zhejiang 325024, China; ^4^School of Material Science and Engineering, Georgia Institute of Technology Atlanta, GA 30332, USA

## Abstract

The importance of ocean exploration and underwater monitoring is becoming vital, due to the abundant biological, mineral, energy, and other resources in the ocean. Here, a self-powered underwater cable-based triboelectric nanogenerator (TENG) is demonstrated for underwater monitoring of mechanical motion/triggering, as well as searching and rescuing in the sea. Using a novel double-layer winding method combined with ferroelectric polarization, a self-powered cable-structured sensor with a stable electrical output has been manufactured, which can accurately respond to a variety of external mechanical stimuli. A self-powered cable sensing network woven using smart cables can comprehensively transmit information, such as the plane position and dive depth of a submersible. More precisely, it can analyze its direction of movement, speed, and path, along with transmitting information such as the submersible's size and momentum. The developed self-powered sensor based on the cable-based TENG not only has low cost and simple structure but also exhibits working accuracy and stability. Finally, the proposed work provides new ideas for future seabed exploration and ocean monitoring.

## 1. Introduction

As a significant treasure house of minerals [1], medicinal materials, biology, and other resources [2], the ocean has attracted significant research attention. With the development of ocean exploration, more precisely the way the position and movement information of the submersible should be transmitted in the dim, topographic, and ecologically complex underwater environment poses a huge challenge to underwater detection technology. Simultaneously, the commonly used detection methods (i.e., the sonar technology [3]) also face challenges, such as the high frequency of sound waves, poor bio-friendliness, low degree of modularity and systemization, high cost, and the requirement of significant maintenance [4, 5]. Therefore, there is an urgent need to develop the novel underwater sensors [6], in order to match the ever-increasing demand for undersea detection, rescue, and defense.

The operation of triboelectric nanogenerator (TENG) is based on the coupling effect of contact electrification and electrostatic induction [7–9], which leads to high performance and high efficiency in collecting tiny mechanical energy and converting it into electrical energy power [10, 11]. It also has the characteristics of simple structure [12–14], environmental protection [15–17], low cost [18–20], wide application [7, 21], and high safety [22]. In addition, this self-powered working mode, converting the external mechanical energy into electrical signals, provides new strategies for the development of small-sized and high-precision sensors [23–27], such as fiber-based or fabric-based self-powered sensors and sensing systems [28–31]. The flexibility and structural diversity conferred by textiles allow TENGs to be used in more complex scenery or under special working conditions [32]. The inexpensive and easily available materials also give them the possibility of large-scale industrial production [30, 33, 34]. As the most basic design unit in modern smart fabrics [34–36], the diameter of fiber-based TENG is usually only a few millimeters or less [37–39]. This macroscopic one-dimensional structure makes the internal structure of the smart fiber extremely difficult to contact the external environment and is not easy to be mechanically damaged or affected by humid and corrosive environments, which provides a high potential to overcome the ocean exploration challenges.

In this study, a self-powered sensing underwater cable based on cable-structured TENG (CS-TENG) which has the best flexibility, practicality, and environmental protection is designed. The electrical output performance of CS-TENG has been significantly improved through material selection [40–42] and structural optimization [43]. Considering that underwater monitoring is triggered by mechanical movement, different signal sources can be easily distinguished. The woven smart network results from the underwater cables integrate the functions used to monitor the submersible's motion trajectory, motion speed, and dive depth.

## 2. Results

**Figure 1:**
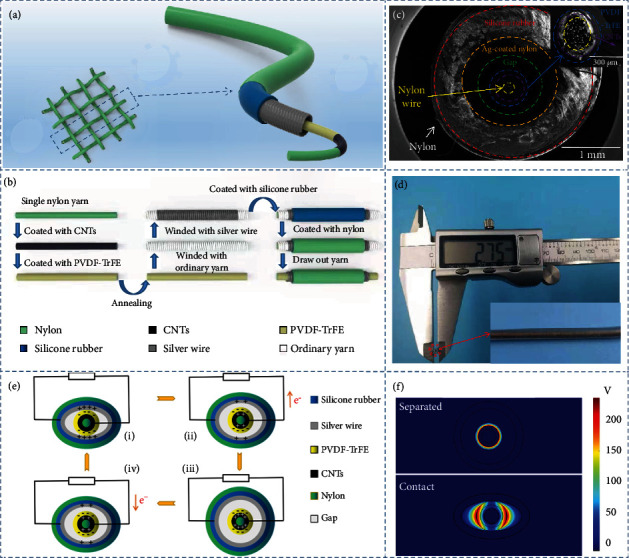
Schematic diagram of the structure, preparation process, and working principle of CS-TENG. (a) Structure diagram of the flexible TENG having underwater working ability. (b) Demonstration of the preparation process of CS-TENG. (c) SEM image of the underwater cable radial cross-section, in which the different coaxial functional layers are distinguished by dotted lines of different colors. (d) The use of a micrometer to measure the diameter of a cable. (e) Charge distribution diagram of CS-TENG in normal operation, under the condition of external resistance. (f) Numerical calculations of the potential distribution of CS-TENG under open-circuit conditions using COMSOL software.

River basins or deep-sea areas have complex geographic environments and ecosystems, while self-powered sensors or systems based on the CS-TENG show significant advantages because of their simple structure and durability. A large quantity of water will carry away the charges generated by the contact surface. Therefore, it is beneficial to package the self-actuated sensor by choosing the vertical contact-separation mode, which leads to enhanced sensitivity and robustness. The coaxial core-shell structure ideally creates a contact-separation space, while the possible contact direction is extended to 360°. In addition, the preparation process is relatively simple and adaptable for mass production. The specific structure is shown in Figure 1(a). The nylon fibers are selected as the base material, due to their high flexibility and breaking strength. Carbon nanotubes (CNTs) are the optimal choice for the core electrodes due to their low price, good electrical conductivity, and elasticity. One of the selected triboelectric materials is organic piezoelectric material poly(vinylidene fluoride-trifluoroethylene) (P(VDF-TrFE)). The other triboelectric material is silver fiber, which is also the shell structure electrode. Increasing the density of silver fiber will not only increase the contact area but also the electrode area. Due to the restriction of the friction force and contact-separation space, it is hard to increase the number of turns of silver fiber. In this paper, a double-layer winding approach is used. As shown in Figure 1(b), a layer of thick fiber is tightly wound around the core structure, followed by a thin silver fiber layer. After encapsulation, the friction force is evenly distributed across the thick fiber. It can be observed from Figure 1(c) that although the outer silver fiber has a high number of turns, the thick fiber can be screwed out without breaking the original structure and creating a contact-separation space between the core and the shell structure. The gap increases with the increase of the thick fiber diameter, but this increasing is limited. More precisely, if the diameter ratio of two fibers exceeds a certain threshold, a gap between the adjacent two turns of the thick fiber will be filled by the silver fiber, hindering the subsequent spin-out of the thick fiber. This issue can be solved using a multilayer winding approach. According to the axial profile of the shell structure (Figure S1), the electrode of the CS-TENG is dense and evenly arranged, which proves that the approach is very reliable and efficient. This is crucial in the preparation of smart fibers that have a complex internal structure and cannot be contaminated. Water-resistant silicone rubber is coated on the shell structure to enhance hydrophobicity and corrosion resistance. The detailed method of preparation is provided in the experimental section. A complete CS-TENG prepared according to the above process is measured with a micrometer, and diameter of CS-TENG is only 2.75 cm (Figure 1(d)).

**Figure 2:**
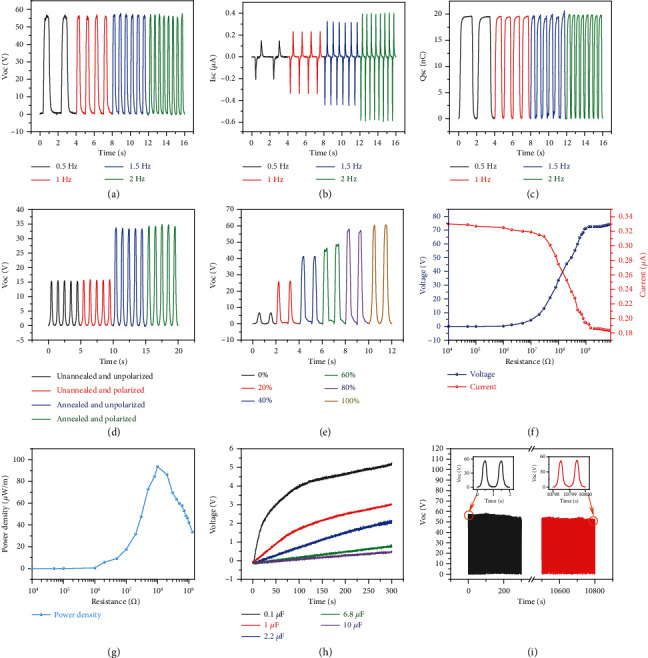
Characterization and modification of underwater electrical output performance of CS-TENG. (a)–(c) Electrical output performance of CS-TENG (length 5 cm) with a constant applied load at different frequencies (0.5-2 Hz), including (a) Voc, (b) Isc, and (c) Qsc. Variations of electrical output performance by optimizing the (d) core structure and (e) shell structure, respectively. (f) Output current and voltage of the CS-TENG connecting with different external resistances. (g) Dependence of the output peak power density on external loads. (h) Charging curves of capacitors with different capacitances (0.1-10 *μ*F). (i) Characterization of CS-TENG cycle life for more than ten thousand cycles.

Figure 1(e) briefly shows the functioning process of CS-TENG. If an external force acts on the outer surface, the shell structure undergoes a compression deformation. The silver fiber gradually approaches and contacts the P(VDF-TrFE). At this time, the free electrons in the silver fiber are transferred to the surface of the P(VDF-TrFE), leading to the charges of the same size but with the opposite sign distributed on both surfaces. Equal amount of positive charges will be generated in the CNTs layer through the electrostatic induction. After removing the external force, the deformation recovery occurs with the separation of the two triboelectric materials. In this process, the charges in CNTs and in the silver wire transfer through the external circuit, thus, completing a cycle. As the contact-separation process occurs multiple times, an alternating current is generated in the external circuit. COMSOL Multiphysics software was used to simulate the electrical potential changes at each part in the process of contact and separation, as shown in Figure 1(f). It can be clearly observed from Figure S2 that P(VDF-TrFE) is annealed at 135°C under vacuum to form the ferroelectric phase *β*. As contact electrification occurs, the positive and negative charges interact to form a microscopic electric field, while the originally disordered electric dipoles in the ferroelectric phase are arranged in an orderly manner in the direction of the electric field lines. The separation of the centers of the positive and negative charges is referred to as ferroelectric polarization. The latter generates a new microelectric field in PVDF-TrFE, which in turn enhances its ability to obtain electrons and eventually leads to an improved output performance (Figure S3). A linear motor and an acrylic tank are combined in order to simulate external underwater excitation. The underwater electrical output performance of CS-TENG is tested and evaluated using a programmable electrometer. This test is conducted at a depth of 10 cm. The temperature and air pressure are kept at 298 K and 101 kPa, respectively. A force of 10 N is maintained along with a frequency of 0.5 Hz for continuous tapping. The maximum distance of each cycle is 20 mm. The output is allowed to become stable, while the Voc, Isc, and Qsc values of the 5 cm CS-TENG can reach ~56.8 V, ~0.21 *μ*A, and~19.6 nC, respectively. When the frequency is gradually increased to 2 Hz, Voc and Qsc do not significantly change, while Isc increases to ~0.58 *μ*A (Figures 2(a)–2(c)). Figure S4 illustrates a test of electrical output performance in air. It can be seen that the obtained results fit with the characteristics of the sensors for underwater work, which operates at a low frequency but requires a high accuracy. It can be observed from Table S1 that the electrical output performance of CS-TENG in the present work is significantly improved, compared with the literature [26, 37, 44, 45]. The electrode area in the control shell structure accounts for 40% of the inner surface area of the package layer. The Voc increase is not significant, after the core structure was polarized in an electric field of 30000 V/m for 5 min. Although the diameter of the core structure is close to a single line, the P(VDF-TrFE) layer is actually a tubular body with a three-dimensional structure. This results in aligning the electric dipoles in the polarization process along the direction of the electric field lines. However, in the actual test, the contact-separation process may occur in any radial direction, and the regularly arranged electric dipoles do not enhance the surface polarization in most directions. This phenomenon is shown in Figure 2(d). Multiple shell structures are manufactured with different winding turns of the silver wire. The core structure is unchanged, and the density of the sheath electrode is expressed by the percentage of the area occupied by the silver wire on the inner surface of the packaging layer. The influence of changing the electrode density on the Voc of CS-TENG was tested. It can be seen from Figure 2(e) that Voc presents an upward trend. However, beyond a certain threshold, the overdense silver fiber also increases the rigidity of the shell structure. Therefore, the deformation degree of shell structure under the same external force decreases, and the upward trend of Voc slows down. Considering the electrical output performance and sensitivity, the shell structure with the silver wire accounting for 80% and the core structure after the annealing treatment is selected. When the external load resistance increases, the voltage increases, while the current undergoes the opposite behavior, which is based on the Ohm's law (Figure 2(f)). Once the resistance value of the external load is close to ~100 M*Ω*, the power output density reaches its peak value of ~95.5 *μ*W·m^−1^ (Figure 2(g)). The relation used to calculate the power output density (*P*) is given by:

(1) $$\begin{array}{c} P=\frac{U^{2}}{R\times a}, \end{array}$$

where *U* is the voltage under the external load, *R* presents the resistance of the external load, and *a* denotes the length of CS-TENG.

At a working frequency of 1 Hz, a capacitor of 1 *μ*F can be charged to ~3 V in 300 s (Figure 2(h)). After cycling for more than 10,000 times, the output voltage is reduced by ~5%, which verifies that CS-TENG is able to continuously work underwater (Figure 2(i)).

**Figure 3:**
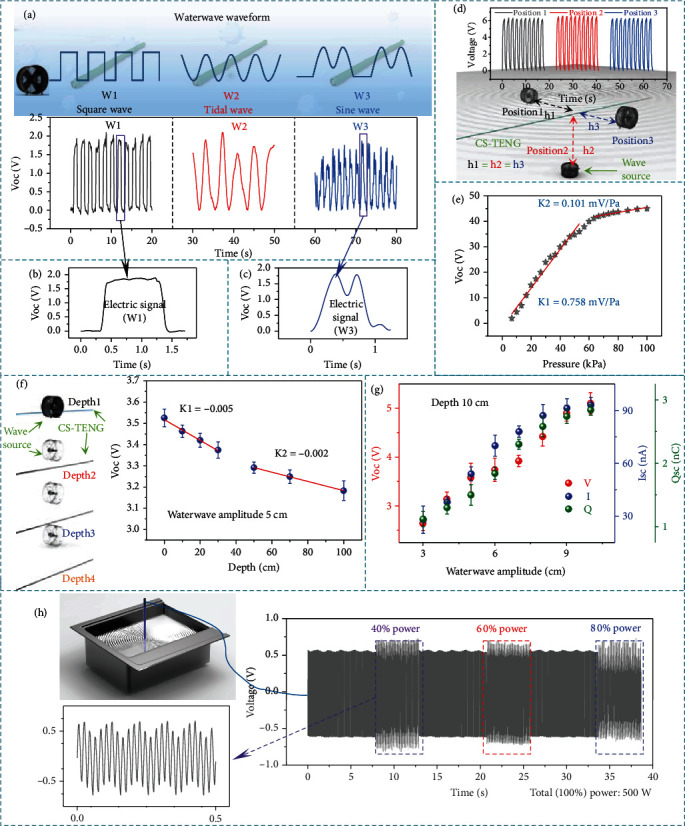
Sensitivity characterization of CS-TENG and its response to ultrasonic waves. (a) Electrical response of CS-TENG to various waveform of water wave, including (b) square wave (W1), tidal wave (W2), and (c) sine wave (W3). (d) Electrical response of CS-TENG to changing the position of the water wave source. (e) Pressure response of the cable to various applied loads. (f) The trend between the change of open-circuit voltage with depth. (g) Electrical outputs of the CS-TENG under different water wave amplitude. (h) Response of the CS-TENG to ultrasonic stimulation at different input power for the sonic wave.

Besides the vertical loads, other loads may also be applied in the axial direction during a practical application. It can be seen from Figure S5 that an increment in the electrode coverage in the shell structure leads to a reinforced fracture strength of the shell structure. The base material in the core structure itself has a high elastic modulus, and the presence of P(VDF-TrFE) coating further optimizes the fracture strength. Consequently, the tensile deformation of the core structure will be much smaller than that of the shell structure when simultaneously applying the same axial load and the vertical contact-separation phenomenon appears between the two triboelectric materials. The electrical output signal caused by the radial stretching is not large. However, the degree of deformation change is more obvious (Figure S6) and durable (Figure S7). This demonstrates that CS-TENG can adapt to the complex mechanical deformation (Figure S8) and also retains its stability even after being immersed in seawater for a long time (Figure S10). As shown in Figures 3(a)–3(c), a wave pump is used to simulate water waves of different waveform, including square waves, tidal waves, and sine waves. CS-TENG shows distinct responses to different water wave stimuli, and the waveform of its voltage signal is roughly the same as that of the water wave, which is of great significance for distinguishing different signal sources. As shown in Figure 3(d), the water wave source was placed at different positions around the CS-TENG and ensures that the relative distance between them is consistent for testing. The almost constant signal peak eliminates the influence of the wave source position change on other tests. When increasing the pressure at 60 kPa, the voltage increases rapidly due to an optimal contact process. Above 60 kPa, the lifting speed is reduced because of the limited contact-separation space (Figure 3(e)). The change in water pressure caused by changes in water depth will also affect the sensor signal. But this kind of pressure is always there, which will cause the distance between the core structure and the shell structure of CS-TENG to decrease. This will cause the output of CS-TENG to decrease with increasing depth under the same pressure. In order to explore the extreme depth of the sensor's precise work, we tested the correlation between the output signal of CS-TENG and the depth in the range of 0-100 cm. Figure 3(f) shows that in this range, control the mechanical pressure exerted on CS-TENG unchanged, the voltage decreases slowly as the depth increases, and the working sensitivity of CS-TENG also decreases as the depth increases. In addition, the ultimate working depth can be calculated based on the fitted curve. The amplitude of the water wave is also one of the factors that have the greatest impact on voltage. As the amplitude increases, the load applied to CS-TENG is also greater. As shown in Figure 3(g), under the condition of keeping the depth constant and the waveform unchanged, the open-circuit voltage, short-circuit current, and the amount of transferred charge all show a clear upward trend with the increase of amplitude. The almost linear trend has also become a strong support for quantitative analysis of underwater targets. The limit wave amplitude and limit the distance of the sensor were tested under the condition of fixed water wave amplitude and the distance between the wave source and the sensor. When the peak value of the obtained signal is less than 0.1 V, since the signal-to-noise ratio is lower than 3, it is not conducive to subsequent waveform recognition. Therefore, it is considered that the limit has been reached (Figure S11). Finally, the response of CS-TENG to ultrasonic excitation of different powers was tested. Due to structural limitations, the response to ultrasonic waves is far weaker than the response to water waves. Figure 3(h) and Figure S12 show that as the ultrasonic power increases, the voltage or current gradually decreases. This is because the internal structure will receive the next vibration before it reaches the maximum amplitude. Based on the energy generated by ultrasonic excitation, a 68 pF commercial capacitor can be charged to 1 V in 60 s (Figure S13).

**Figure 4:**
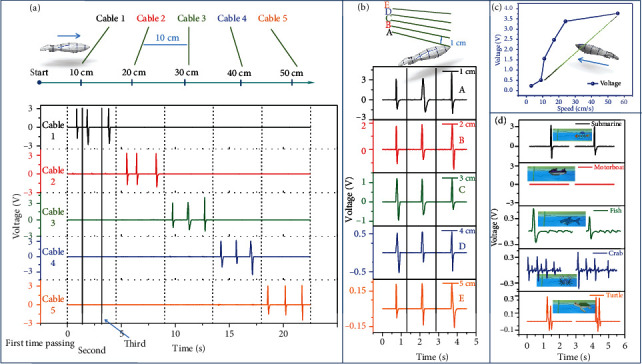
Underwater position monitoring of CS-TENG. (a) A group of underwater cables arranged in horizontal gradient to monitor the approximate position and average speed of underwater objects. (b) A group of underwater cables arranged in the depth gradient to monitor the diving depth of underwater objects. (c) A single cable sensor for monitoring the submersible instantaneous speed. (d) Comparison of the sensor signals caused by different biological and man-made objects passing through the cable.

Existing methods for monitoring of underwater vehicles and searching for submersibles are mainly based on the sonar detection. However, the widespread use of electromagnetic shielding materials allows submersibles to be invisible. The invisible nature of the submersible only hinders detection based on sound waves, while the directional water flow caused by volume and operation cannot disappear. As shown in Movie S1, due to their mechanical movement, the monitoring function based on CS-TENG is triggered. Many cables are horizontally arranged in the ocean so that the spacing between them is greater than the average length of the submersible. When a submersible navigates above or below one of the cables, it has a small effect on the rest of the cables in the array. It can be seen from Figure 4(a) that, compared with the submersible, the underwater cable is minimal in the radial direction and can be considered as one dimensional. Therefore, compared with the submersible with a larger momentum and volume, it can accurately reflect the moving index of the passing submersible while maintaining its concealment. The submersible's diving depth is a difficult indicator to be monitored. This is due to the fact that it consists of taking in and removing water, which is totally different from moving forward. In order to overcome this issue, several cables are arranged in parallel to the same vertical plane under the water, according to a certain height gradient. It can be observed from Figure 4(b) that when a deep-diving submersible passes through this vertical plane, the peak value decreases as the vertical distance between the cable and the submersible increases. Assuming that one of the cables is damaged due to the impact of submersibles, creatures, or other accidents, the whole system can still remain stable. In addition, in actual waters, when a submersible stays at a certain location, the subsequent cables in the array will no longer generate signals. Therefore, it is very important to identify and explore this location. A method of literature is presented in Figure 4(c). The correlation between the speed of the submersible and the output voltage was tested. So that the submersible's instantaneous velocity can be roughly estimated by one-to-one to correspondence. An average velocity can be obtained when the submersible passes through a parallel array of cables, which combining with the instantaneous velocity, enabling the acceleration and approximate position prediction. Fortunately, these complex calculations and comparison processes can be solved using a specially designed signal processing module. The difference in signal peak value is caused not only by the differences in velocity but also by the mass. In other words, a positive correlation exists between the signal peak and the momentum. Therefore, if the velocity does not change much, the submersible's mass can be inferred based on the corresponding relationship between the signal peak value and the momentum, then, the type of submersible (or what it carries) may be analyzed. Note that, besides the submersibles, yachts and other man-made entities are present in the ocean. The signal caused by them is unavoidable. For this reason, some models that highly simulate the movement of the marine organisms are used to simulate the real scenario, as shown in Figure 4(d). A motorboat floats on the water and relies on the propeller to move forward. This kind of motion mode will highly enhance its speed, due to the reduction of the water resistance, and the waves will be concentrated on the surface. It is hard to generate signals using the cables which work in large depths when a motorboat passes quickly over them. From the perspective of the direction acting on the sensor, the amplitude of the water wave generated by the motorboat does not reach the minimum amplitude measured by the sensor. Therefore, this mechanical movement does not trigger the monitoring effect. Unlike submersibles, marine organisms generally cause multiple signal peaks. The energy range that can be captured by the underwater cable is assumed to be a space. When the fish first touches the space, a larger peak is generated. Subsequently, due to the swing of the fin and tail, it will bring several continuous signal fluctuations of small amplitude (Movie S2). The crab's several pairs of feet are continuously curled and stretched when swimming. When passing through this space, the stretched feet triggers a larger peak, while the curled feet cause a small amplitude signal to float. The turtles move very fast, however, they can also be divided into forelimb and hindquarters strokes, where each forelimb or hindquarters stroke triggers a corresponding peak. However, whether this way of distinguishing waveforms is completely triggered by mechanical motion is a crucial question. Especially when the distance between the monitored object and the sensor is very close, if the electrical signal has surface dependence, it will interfere with the subsequent analysis and judgment. As shown in Figure S14 and Movie S3, after different surface treatments are performed on the test model, the experiment of triggering the monitoring mechanism is performed with the same conditions. After different surface treatments, there is almost no change in electrical signals, which shows that the monitoring mechanism of CS-TENG is completely triggered by mechanical motion. Such waveform-based judgments are valuable. However, they are not entirely reliable. For instance, cephalopods (such as octopuses) act as “rockets under the sea” as they move forward, adding to the difficulty of distinguishing them from man-made objects such as submersibles. This requires comparing the signals from different cables. Therefore, it is suggested using self-powered sensor networks woven from CS-TENGs in order to achieve a high degree of intelligence, systematization, and function integration.

**Figure 5:**
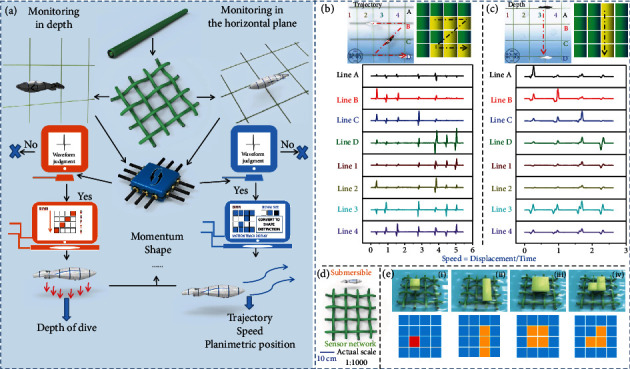
Principle of positioning and multifunctional monitoring of underwater cable network. (a) Real-time monitoring concept map of the underwater objects is realized through the joint action of the vertical and horizontal networks. (b) Vertical and horizontal networks used to obtain the motion trajectory and (c) diving depth of underwater objects, and combined with time to output the speed information. (d) The size of the experimental device and the scale of the experimental device to the real object in the real scene. (e) Real-time monitoring of important information, such as the (i) momentum, (iii) size, and (ii-iv) shape of underwater objects.

Multiple underwater cables are interwoven according to the way of warp and weft, in order to form a sensor network. Based on the LabVIEW software platform, the signal differentiation and the processing module are particularly designed to coordinate with the multichannel signal acquisition module (Figure 5(a)). A small acrylic block is used to simulate a submersible with a certain momentum and volume, as shown in Figure 5(b). The changes in the point of action or impact force caused by changing the diving depth or changing the horizontal motion are captured by the horizontal sensor network. This change will be intuitively presented in the form of color block changes. The change of position composes the motion trajectory graph. This can monitor the moving state of the submersible in a certain plane in real-time (Movie S4). Based on this positioning method, it is also possible to monitor the average movement speed of the submersible. The displacement information (*x*) and time information (*t*) are reflected by the motion trajectory, representing the sufficient conditions required to obtain the motion speed (*v*):

(2) $$\begin{array}{c} v=\frac{x}{t}. \end{array}$$

The horizontal network is used to perceive changes in the dive depth, following the same functioning principle of the vertical network (Figure 5(c)). This positioning method, which integrates the monitoring of the submersible's movement direction, diving depth, speed, and other information, is more specific and accurate than the positioning achieved by using the cable array arranged in parallel. In actual operations, search and rescue or interception will also be more precise, and therefore, saving manpower and material resources. At the same time, in order to accelerate the application of this CS-TENG-based underwater sensor network to the actual process, the scale between the model used in the test and the actual object is shown in Figure 5(d). This operating mode is not limited to the detection of the position. The signal identification and processing modules are optimized with new algorithms so that the self-powered sensing system also has the function of detecting the shape, size, and momentum of the object (Figure 5(e) and Movie S5). Combined with the monitored shape and size, a rough outline of the underwater object can be obtained, which is not achieved by a single cable or a parallel cables array. It is very likely to make contributions to the gradual visualization and transparency of the mysterious deep-sea area. An object with a large momentum will cause a larger voltage signal. When the voltage exceeds a certain threshold, the program will display the object's action point in red (Figure S15). If the cable is destroyed due to the impact force, the self-powered sensor network will no longer carry out waveform discrimination, which triggers the emergency red warning signal. The system provides a high level of safety, with timely stop-loss and minimization of the potential risk.

This research is different from traditional underwater monitoring devices. A major feature of TENG-based sensors is self-powered operation. Especially in the deep sea environment, it is very difficult to replace the battery of the sensor, which will cause a lot of waste of resources. At the same time, the monitoring function is triggered by the monitored object, so there is no need to send additional artificial waveform. This makes the information reflected by the obtained signal completely the motion information of the monitored object and also avoids the attenuation of the signal in the water. This new underwater monitoring method is not only more environmentally friendly, saves resources, is conducive to achieving carbon neutrality, but also transmits more accurate information.

There are still some issues that need to be considered in depth when this research work is put into real applications. The monitoring function of the underwater sensor network is based on the response of CS-TENG to water waves. In the real seabed environment, tides, undercurrents, creatures, etc. all produce water waves. There may be multiple objects on the seafloor that trigger the monitoring function at the same time. Solving this type of problem may require combining deep learning technology or other auxiliary detection technologies. For monitored objects such as submersibles, they may not move according to a certain pattern, or even stagnate somewhere. In addition, the shape of the detected object may also change, such as extending the working clamp. This also poses new challenges for underwater monitoring. It will be an effective solution to refine the monitoring function by adjusting mesh spacing appropriately.

The sensor network has a simple structure that is integrated with several functions. It is able to perform timely detection and replacement of the damaged units. Finally, we believe that the underwater cable sensing networks based on TENGs will play an important role in solving the ocean exploration and monitoring issues.

## 3. Conclusion

In this paper, an underwater monitoring network based on CS-TENGs for underwater detection and real-time monitoring is proposed and developed. A peak power density of ~95.5 *μ*W•m^−1^ for a 5 cm-long cable is achieved through the coupling effect of the surface polarization and ferroelectric polarization, using the double-layer winding method. The proposed network based on CS-TENGs has a stable underwater output with a high sensitivity and flexibility. The submersible triggers underwater monitoring through mechanical movement, and thus, its movement direction, speed, and trajectory is collected. This crucial information provides support for further search and rescue/interception. It possesses high modularizaion, systematization, integration, and antidestructive ability. Finally, the proposed detection method has an important reference value and significance for the future development of ocean detection and underwater real-time monitoring.

## 4. Experimental Section

### 4.1. Materials

The nylon fiber and silver-plated nylon fiber were supplied by BOYIN. Ecoflex supersoft silicone (0030) was manufactured by Smooth-On, Inc. N, N-dimethylformamide (DMF), P(VDF-TrFE) powder, nylon powder, and formic acid was supplied by Aladdin Reagent Co. Ltd. Sodium chloride (NaCl) and ultrapure water (H_2_O) were used to configure a 35% salinity solution in order to simulate the sea water.

### 4.2. Fabrication of CS-TENG

#### 4.2.1. The Core Structure

The nylon fiber is first cleaned by ultrasonication in ethanol and deionized water for 15 min. 0.02 g CNT sample is then added to 9.98 g N, N-dimethylformamide (DMF) and subjected to ultrasonic mixing for 30 min, in order to uniformly disperse CNTs in DMF. The nylon yarn is dipped in the CNTs dispersed solution for 10 min, followed by air for other 10 min. The dip coating process is repeated seven times. 2.05 g P(VDF-TrFE) is mixed in 10 g of DMF and stirred at 60°C for 2 h. After cooling, it is successively coated on the fiber five times, followed by air-drying at 80°C for 5 h, and annealing at 135°C under vacuum.

#### 4.2.2. The Shell Structure

The general roving fiber is tightly wound around the core structure. The fine silver fiber is subsequently tightly wound around the general fiber. Afterward, it is placed in a polypropylene tube and then injected with Ecoflex silicone rubber, followed by peeling the tube after curing. The fiber is covered with nylon to enhance hydrophobicity. Finally, the general fiber is spiraled out, in order to generate a contact-separation space. Finally, this leads to the construction of CS-TENG.

### 4.3. Fabrication of the Underwater Cable Sensing Network

The underwater sensor network is composed of CS-TENGs, which is arranged longitudinally and latitudinally, respectively. The tail of CS-TENG is wrapped with hot melt adhesive, and the lead wire electrode is covered with nylon.

### 4.4. Characterization and Measurement

The surface morphology and cross-section of CS-TENG are characterized by AW31 optical microscope and Nova Nanosem 450 field emission scanning electron microscope. The diameter of CS-TENG is measured using a vernier caliper with a high precision digital display. A linear motor (LINMOT E1100) combined with a vacuum grade sealed acrylic tank is used to test the underwater output performance of CS-TENG function of the frequency and force. A compression dynamometer (Vernier LabQuest Mini) is used to measure the applied forces. The open-circuit voltage, the short-circuit current, as well as the amount of transferred charges and sensing electrical signals are measured using a programmable electrometer (Keithley, Model 6514). The software is built based on the LabVIEW platform. The tensile properties are tested using an electronic tensile testing machine (PARAM XLW (B)). The submarines, motorboats, and fish are simulated by the remote-control models, in order to simulate the real scenes. The acrylic tanks are used to simulate the ocean or river basins.

## Data Availability

All data needed to evaluate the conclusions in the paper are present in the paper and/or the Supporting Information. Additional data related to this paper may be requested from the authors upon reasonable request.

## Supplementary Materials

Supplementary Materials Table S1: comparison with fiber-based TENGs mentioned in other works. Movie S1: monitoring whether there is a submersible passing. Movie S2: comparison of sensing signals as different objects pass by. Movie S3: change the “surface” of the monitored object. Movie S4: use the network to determine the underwater position. Movie S5: monitor the size, shape, and weight of underwater objects. Figure S1: axial section view of the shell structure. Figure S2: XRD characterization of P(VDF-TrFE) before and after annealing treatment under vacuum condition of 135°C for 2 h. Figure S3: schematic diagram of the coupling process of ferroelectric polarization and surface polarization. Figure S4: characterization of the electrical output performance of CS-TENG at different tapping frequencies in the air, including Voc, Isc, and Qsc. The tapping force is 10 N, and the length of the cable is 3 cm. Figure S5: stress-strain curves of shell structures with different densities of silver fibers. And stress-strain curve of the core structure before and after being coated with P(VDF-TrFE). Figure S6: the change of open circuit voltage with strain when different axial loads are applied to CS-TENG. Figure S7: cycle life of CS-TENG under axial load. Figure S8: CS-TENG is a graph of open circuit voltage change when the same radial load is applied under different tensile conditions. (The tapping frequency and tapping force are constant at 1 Hz and 10 N.) Figure S9: the underwater cable responds to varying degrees of applied loads. Figure S10: salt corrosion resistance test. Figure S11: sensor detection limit test based on CS-TENG. Figure S12: response of the CS-TENG (current) to ultrasonic stimulation at different input power for the sonic wave. Figure S13: charging curves of capacitors with different capacitance. Powered by ultrasonic energy collected by CS-TENG. Figure S14: the effect of changing the “surface” of the monitored object on the sensor signal. Figure S15: real-time monitoring of important information, such as the momentum, size, and shape of underwater objects. Figure S16: real-time monitoring of human hand and upper limb movement status, including (a) finger bending, (b) changes in the state of fingers when making a fist, (c) changing in the state of palms when making a fist, (d) wrist shaking, (e) elbow bending, and (f) finger pressing. Figure S17: the self-powered sensor based on CS-TENG is used to monitor the motion state of the underwater human body in real time, including finger bending, wrist shaking, making a fist, and bending elbow.
