# Supplementary material for: Underwater Monitoring Networks Based on Cable-Structured Triboelectric Nanogenerators
Source: Research (Wash D C). 2022 Feb 3;2022:9809406. doi: 10.34133/2022/9809406 (PMC8837904; doi:10.34133/2022/9809406)
Supplement: Supplementary Materials — Table S1: comparison with fiber-based TENGs mentioned in other works. Movie S1: monitoring whether there is a submersible passing. Movie S2: comparison of sensing signals as different objects pass by. Movie S3: change the “surface” of the monitored object. Movie S4: use the network to determine the underwater position. Movie S5: monitor the size, shape, and weight of underwater objects. Figure S1: axial section view of the shell structure. Figure S2: XRD characterization of P(VDF-TrFE) before and after annealing treatment under vacuum condition of 135°C for 2 h. Figure S3: schematic diagram of the coupling process of ferroelectric polarization and surface polarization. Figure S4: characterization of the electrical output performance of CS-TENG at different tapping frequencies in the air, including Voc, Isc, and Qsc. The tapping force is 10 N, and the length of the cable is 3 cm. Figure S5: stress-strain curves of shell structures with different densities of silver fibers. And stress-strain curve of the core structure before and after being coated with P(VDF-TrFE). Figure S6: the change of open circuit voltage with strain when different axial loads are applied to CS-TENG. Figure S7: cycle life of CS-TENG under axial load. Figure S8: CS-TENG is a graph of open circuit voltage change when the same radial load is applied under different tensile conditions. (The tapping frequency and tapping force are constant at 1 Hz and 10 N.) Figure S9: the underwater cable responds to varying degrees of applied loads. Figure S10: salt corrosion resistance test. Figure S11: sensor detection limit test based on CS-TENG. Figure S12: response of the CS-TENG (current) to ultrasonic stimulation at different input power for the sonic wave. Figure S13: charging curves of capacitors with different capacitance. Powered by ultrasonic energy collected by CS-TENG. Figure S14: the effect of changing the “surface” of the monitored object on the sensor signal. Figure S15: real-time monitoring of imp [file 9809406.f1.zip › Supporting Information without highlights.docx]

**Supporting Information**

**Underwater monitoring networks based on cable-structured triboelectric nanogenerators**

Yihan Zhang^1,2^, Yingying Li^1^, Renwei Cheng^1,2^, Shen Shen^1^, Jia Yi^1^, Xiao Peng^1,2^, Chuan Ning^1,2^, Kai Dong^1,2,*^ and Zhong Lin Wang^1,2,3,4,*^

1. CAS Center for Excellence in Nanoscience Beijing Key Laboratory of Micro-Nano Energy and Sensor, Beijing Institute of Nanoenergy and Nanosystems, Chinese Academy of Sciences, Beijing 101400, P.R. China

2. School of Nanoscience and Technology, University of Chinese Academy of Sciences Beijing 100049, P.R. China

3. CUSTech Institute of Technology, Wenzhou, Zhejiang, 325024, China.

4. School of Material Science and Engineering, Georgia Institute of Technology Atlanta, GA 30332, USA.

* Corresponding authors:

[dongkai@binn.cas.cn](mailto:dongkai@binn.cas.cn) (K. Dong), [zlwang@gatech.edu](mailto:zlwang@gatech.edu) (Z. L. Wang).

**Table S1. Comparison with fiber-based TENGs mentioned in other works**

| Ref. | Modes | Electrodes | Triboelectric materials | Structures | Power outputs | Power density |
| --- | --- | --- | --- | --- | --- | --- |
| ^[1]^ | SE | CNT/Ag NWs | PDMS  skin | core-shell | 22V  0.21μA  7.4nC | 21.5μW·m^-1^  (150MΩ) |
| ^[2]^ | CS | Bamboo fiber/Ag NW | PDMS  Nylon | core-shell | 3.22V | 1.3 mW·m^-2^  (500 MΩ) |
| ^[3]^ | CS | Ag NWs  PU-Ag NWs | PTFE | core-shell | 0.66V  15nA | 2.25 nW·cm^-2^  (500 MΩ) |
| ^[4]^ | SE | Carbon fiber | Silicone rubber | core-shell | 42.9V | 1.12 μW  (2.5Hz) |
| This work | CS | CNTs  Silver fiber | Ag NWs  P(VDF-TrFE) | core-shell | 56.8V  0.21μA  19.6nC | 95.5 μW·m^-1^  (100 MΩ) |

**Movie S1:** Monitoring whether there is a submersible passing.

**Movie S2:** Comparison of sensing signals as different objects pass by.

**Movie S3:** Change the “surface” of the monitored object.

**Movie S4:** Use the network to determine the underwater position.

**Movie S5:** Monitor the size, shape and weight of underwater objects.

**Figure note 1.** The shell structure is prepared by the double-layer winding method. The silver fiber covers almost all the inner surface of the shell structure, which will greatly increase the contact area and conductivity, thereby optimizing the electrical performance of CS-TENG.


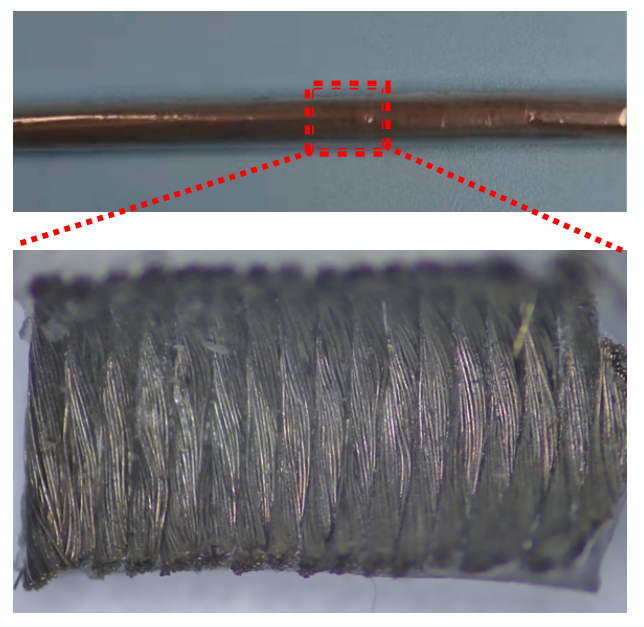


**Figure S1.** Axial section view of the shell structure.

**Figure note 2.** As the annealing process progresses, the peak value of the α phase continues to decrease, while the peak value of the β phase continues to increase. This shows that after annealing treatment, the α phase in P (VDF-TrFE) gradually transforms into β phase.


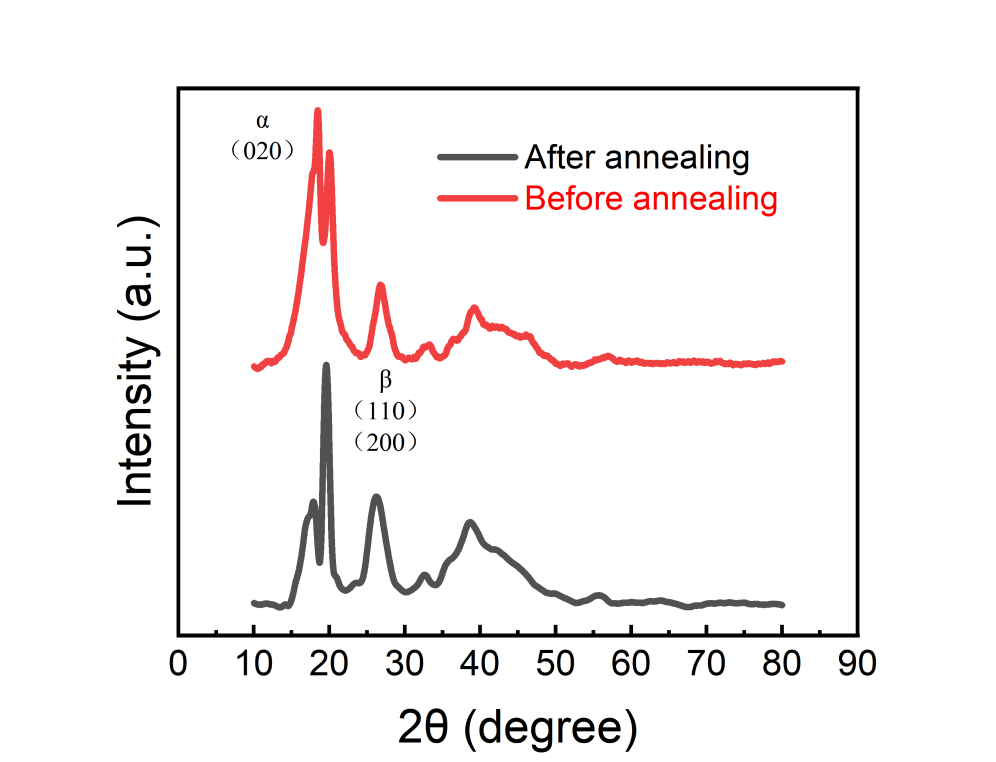


**Figure S2.** XRD characterization of P(VDF-TrFE) before and after annealing treatment under vacuum condition of 135℃ for 2h.

**Figure note 3.** After annealing, P(VDF-TrFE) forms a ferroelectric phase. During the contact-separation process, the transferred charge forms an electrostatic field, which induces polarization. The separation of the center of gravity of the positive and negative charges caused by the ferroelectric polarization creates an electrostatic field, which in turn enhances the ability of the dielectric layer to obtain electrons.


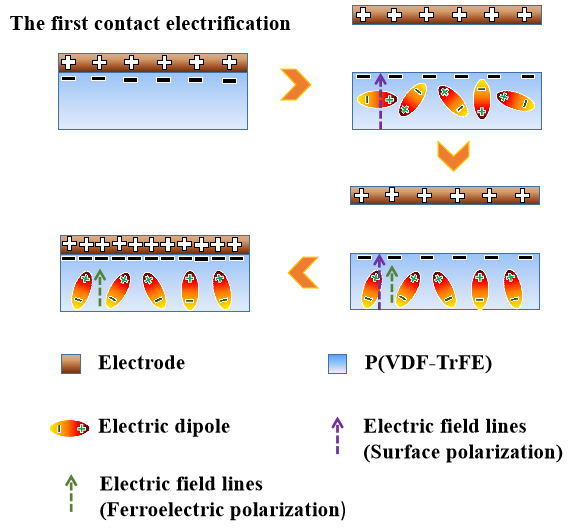


**Figure S3.** Schematic diagram of the coupling process of ferroelectric polarization and surface polarization.


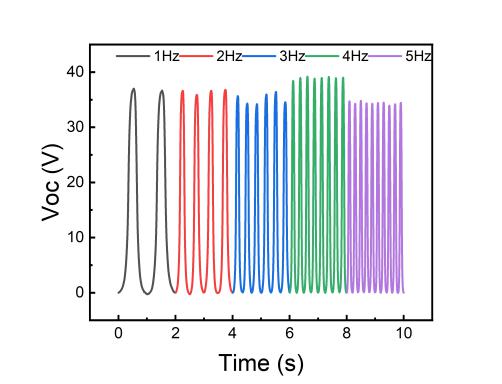

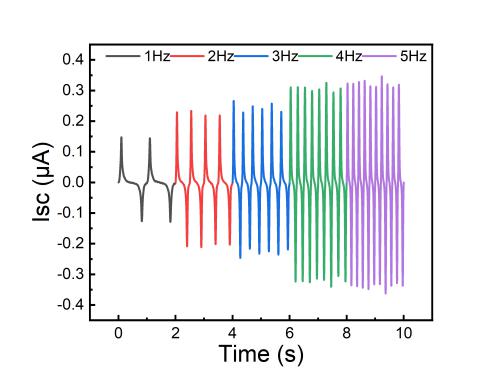

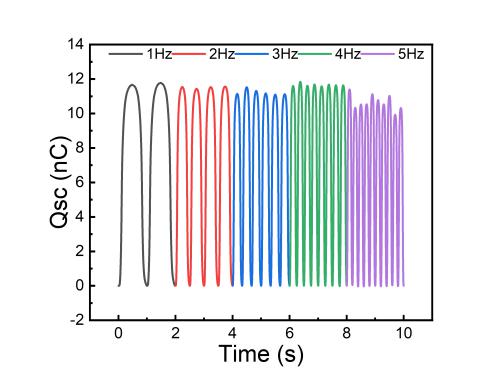


**Figure S4.** Characterization of the electrical output performance of CS-TENG at different tapping frequencies in the air, including Voc, Isc and Qsc. The tapping force is 10N, and the length of the cable is 3cm.


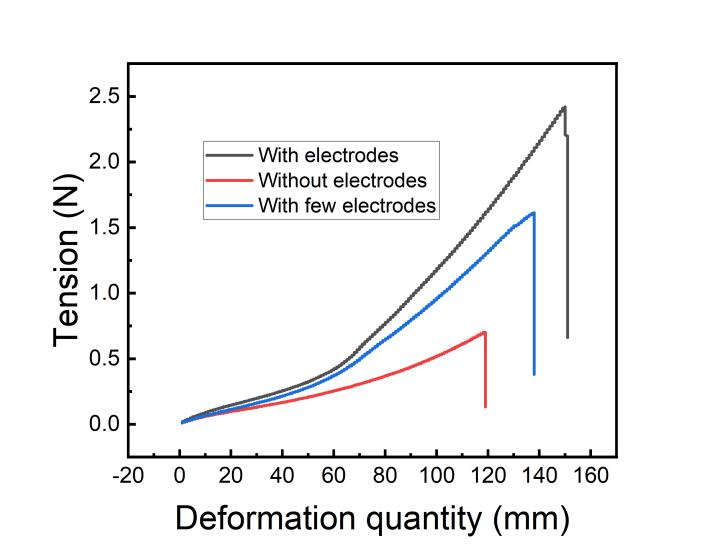

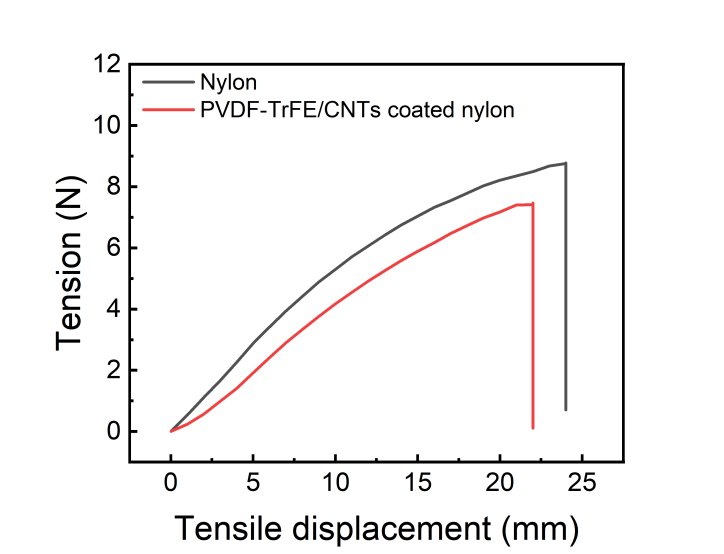


**Figure S5.** Stress-strain curves of shell structures with different densities of silver fibers. And stress-strain curve of the core structure before and after being coated with P(VDF-TrFE).


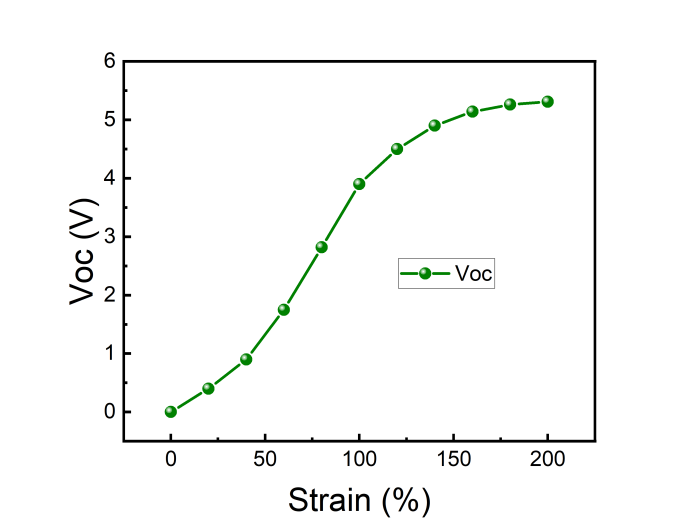


**Figure S6.** The change of open circuit voltage with strain when different axial loads are applied to CS-TENG.

**Figure note 4.** When an axial load is applied to the CS-TENG to cause its axial deformation, then the load is removed to restore it. Repeating the above cycle more than 10,000 times, the attenuation of the open circuit voltage is within 5%, which proves that CS-TENG has a good tensile service life.

**
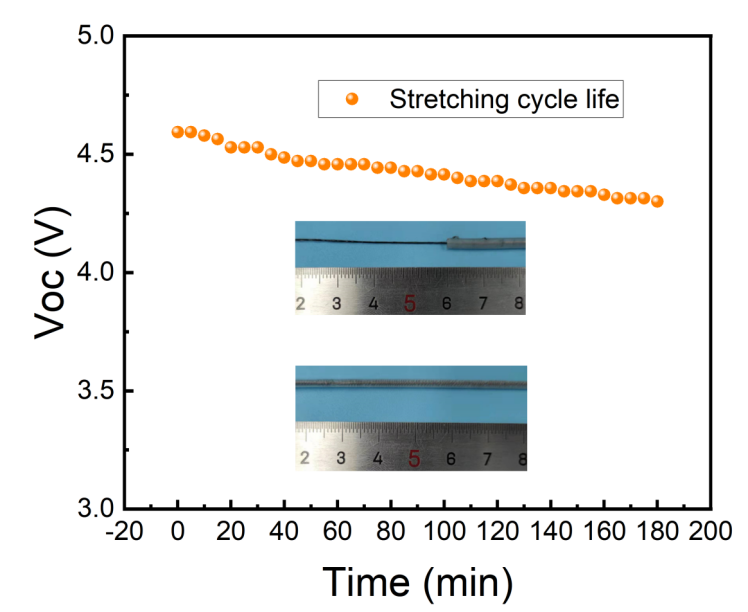
**

**Figure S7.** Cycle life of CS-TENG under axial load.

**
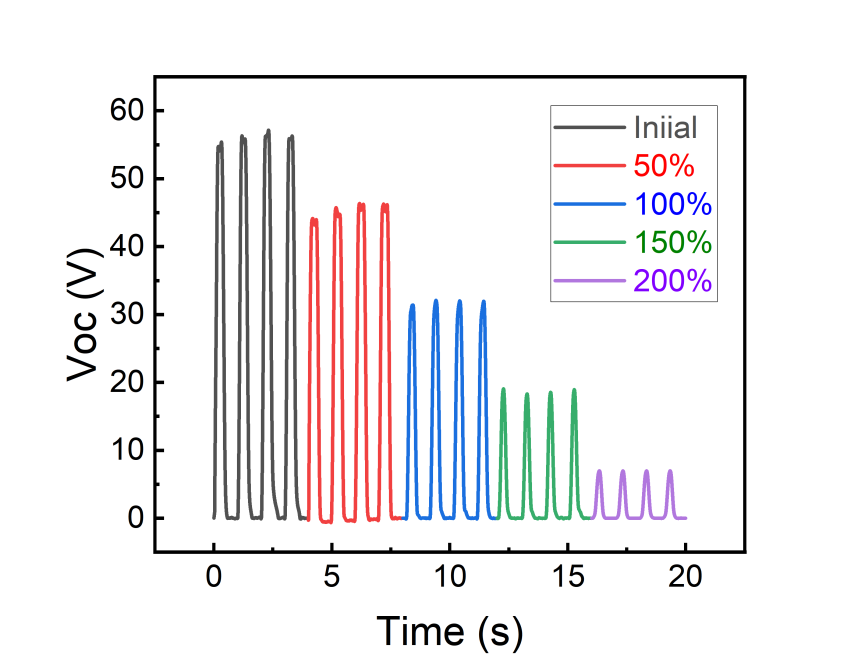
**

**Figure S8.** CS-TENG is a graph of open circuit voltage change when the same radial load is applied under different tensile conditions. (The tapping frequency and tapping force are constant at 1Hz and 10N.)


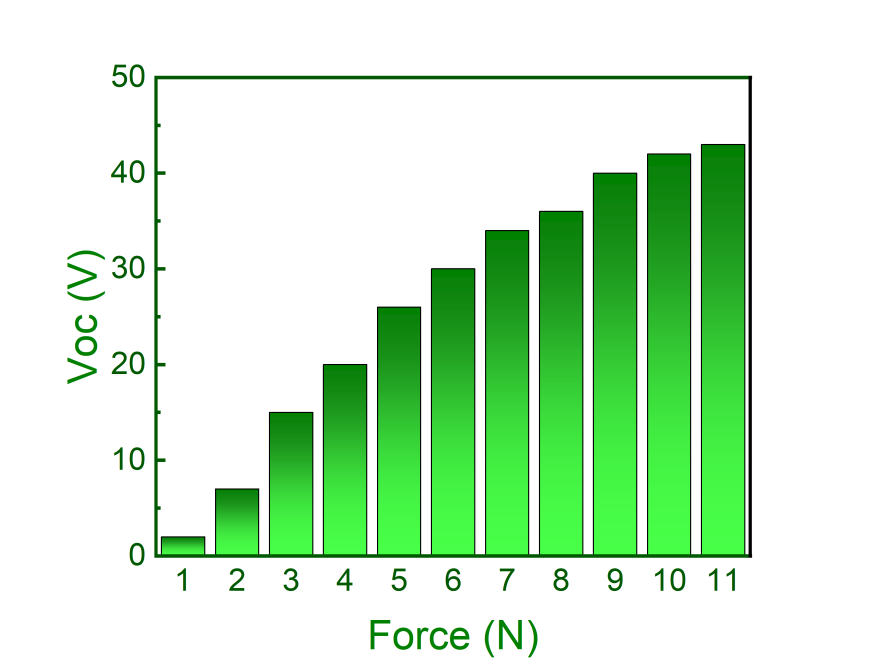


**Figure S9.** The underwater cable responds to varying degrees of applied loads.

**Figure note 5.** Soak CS-TENG in sodium chloride solution (salinity 35%) for 72h, and test its underwater electrical output performance at intervals. It was found that Voc decreased slightly within 12 hours, which was caused by the gradual wetting of the surface. Afterwards, the Voc will hardly decrease, which proves that it has better corrosion resistance.

**
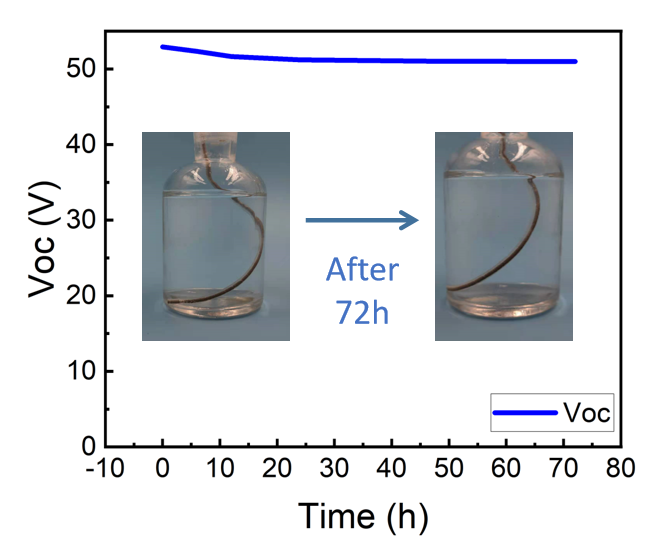
**

**Figure S10.** Salt corrosion resistance test.

**Figure note 6.** The upper left figure is the detection limit distance test, keep the amplitude of the water wave at 3cm and change the distance between the object and the sensor. The upper right figure is the detection limit wave amplitude test, keep the distance between the object and the sensor at 30cm, and change the amplitude of the water wave from the distance. The figure below shows the electrical response curve of CS-TENG at the maximum detection distance and the minimum detection distance. The embedded figure is the change trend of the signal-to-noise ratio when only the distance is changed. When the distance between the object and the sensor reaches 1.1m or more, because the signal-to-noise ratio is less than 3, although the peak value can be observed, the wave change cannot be well recognized, so it is considered that the limit distance has been reached. When only changing the amplitude, the same is true when the amplitude is below 1cm.


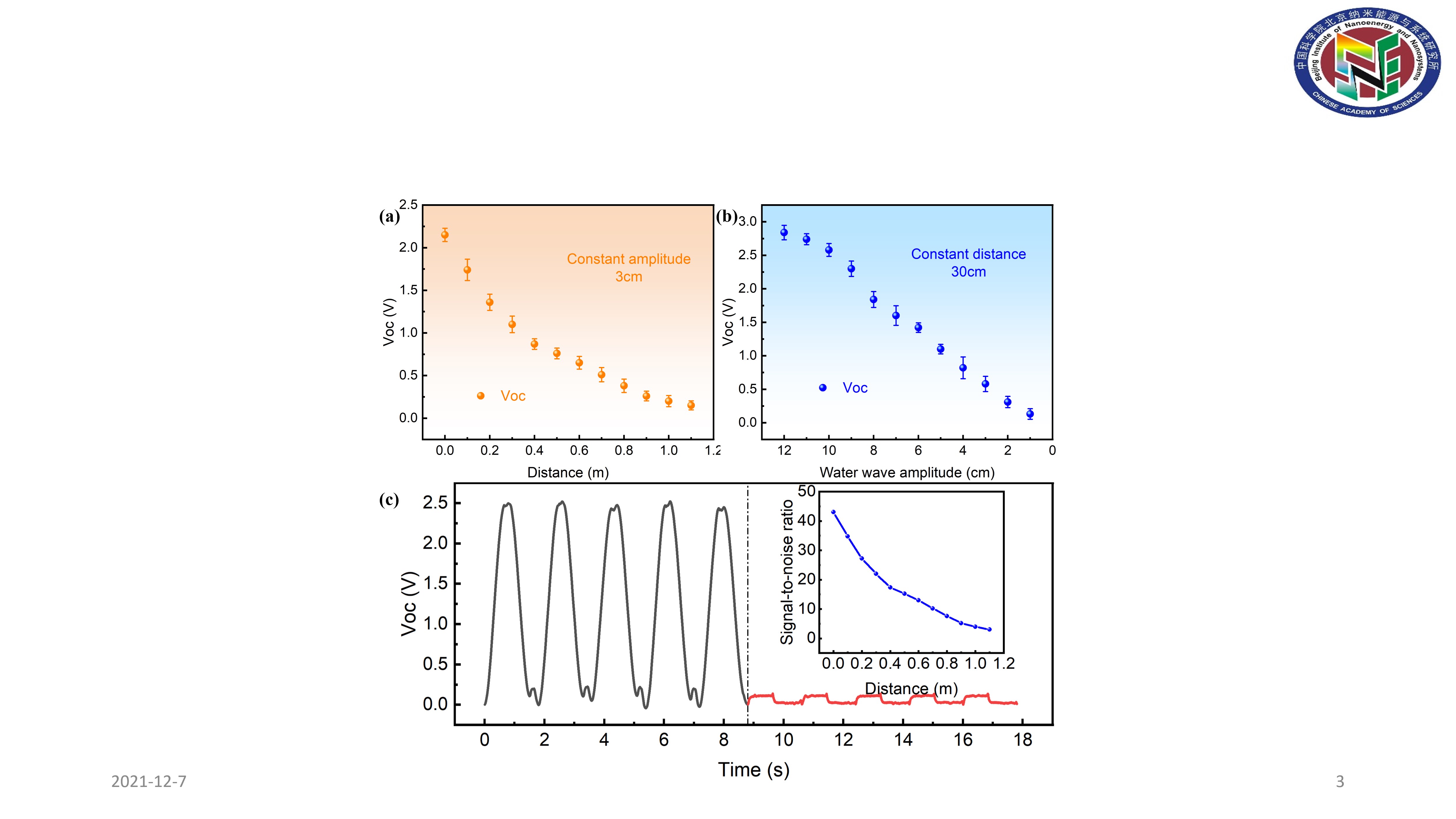


**Figure S11.** Sensor detection limit test based on CS-TENG.

**Figure S12.** Response of the CS-TENG (Current) to ultrasonic stimulation at different input power for the sonic wave.


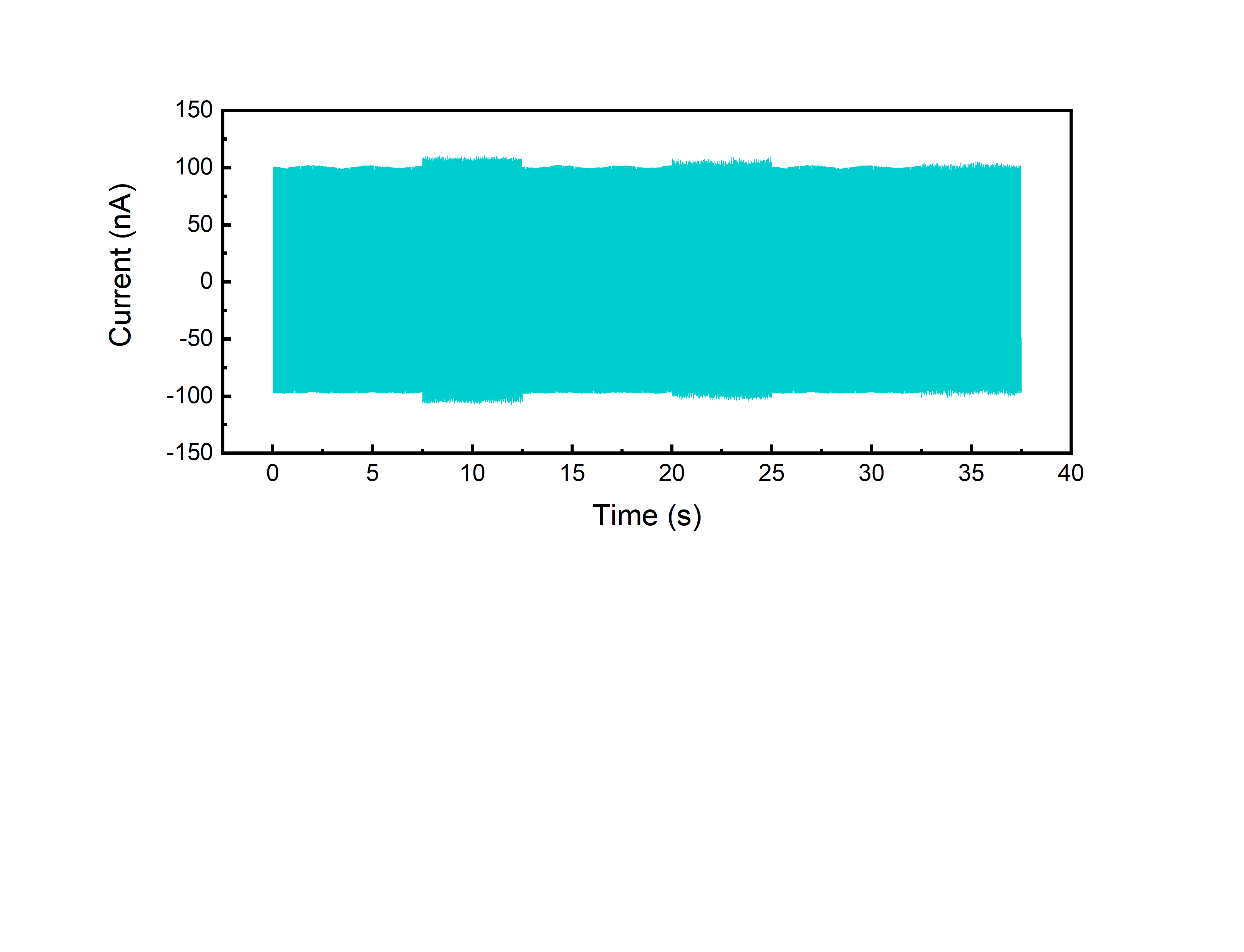


40% power

60% power

80% power

Total (100%) power: 500W


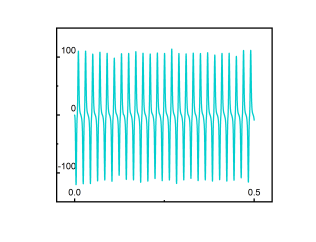


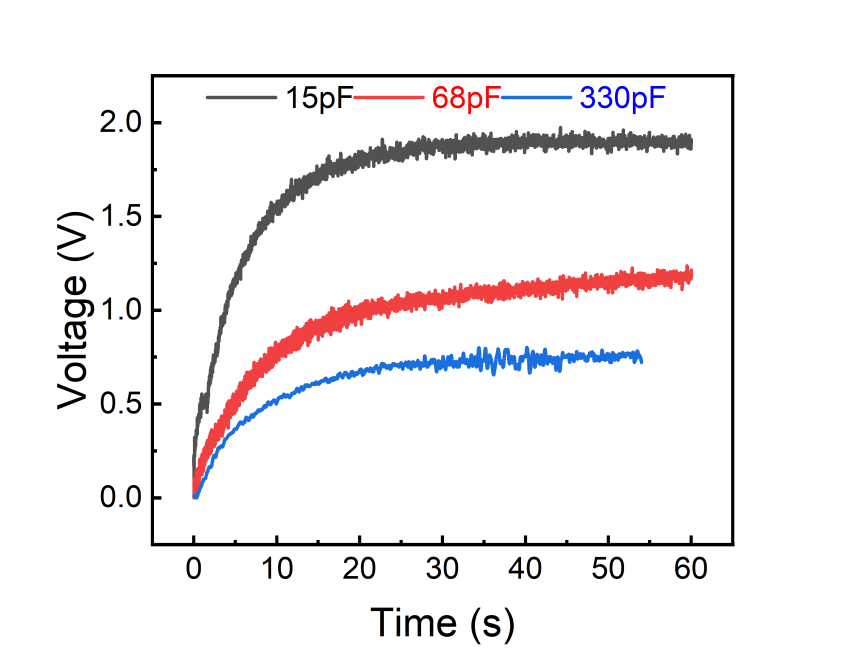


**Figure S13.** Charging curves of capacitors with different capacitance. Powered by ultrasonic energy collected by CS-TENG.

**Figure note 7.** Different treatments (covered with polypropylene (PP) or copper (Cu)) on the surface of the underwater monitoring object, and found that the monitoring signal of CS-TENG has not changed significantly, which proves that this monitoring mechanism is triggered by mechanical motion and is related to the motion of the monitored object. The state is closely related and has little to do with its "surface". The slight decrease in peak value is due to the reduction of the impact velocity by the coating.


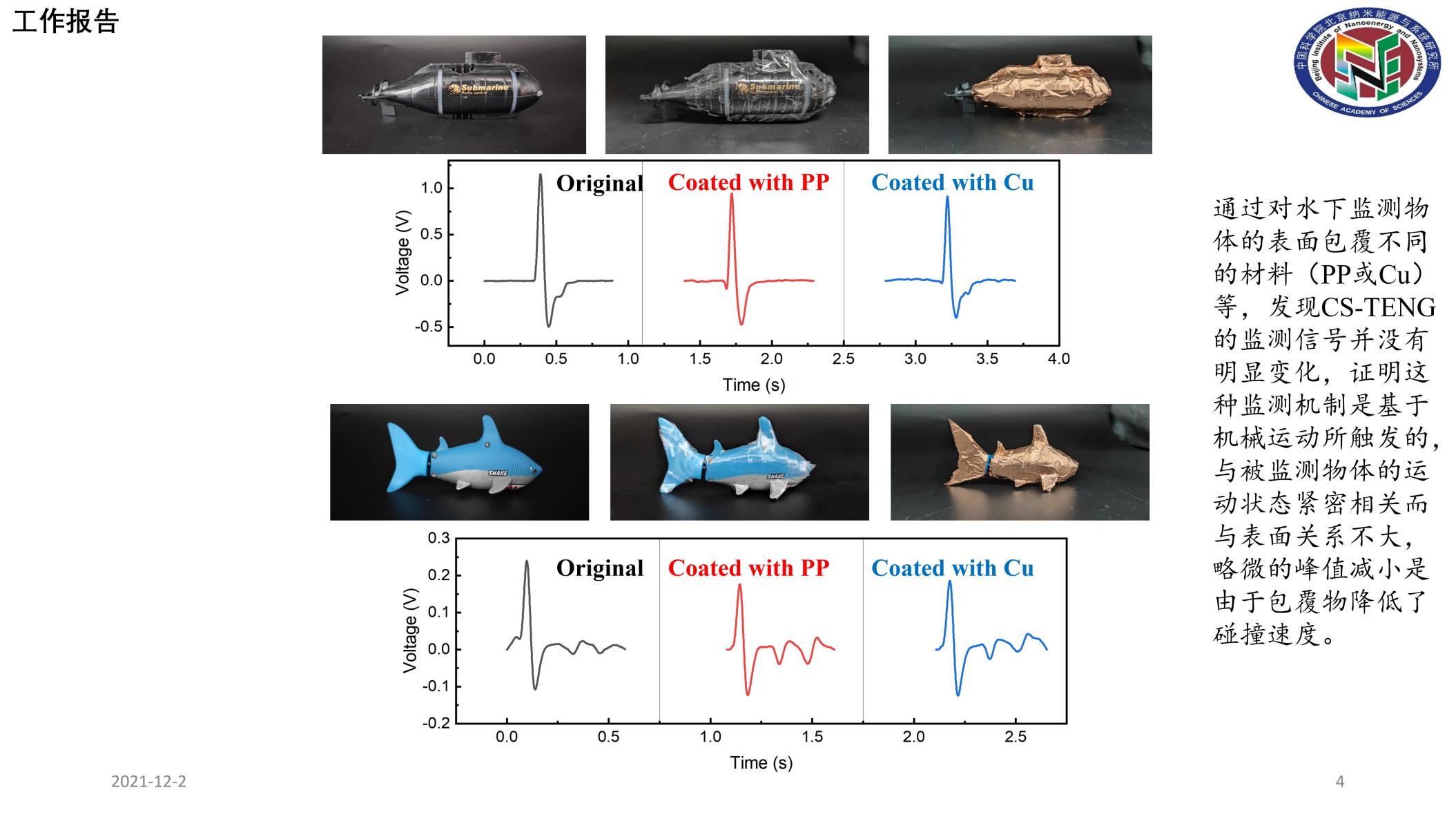


**Figure S14.** The effect of changing the "surface" of the monitored object on the sensor signal.


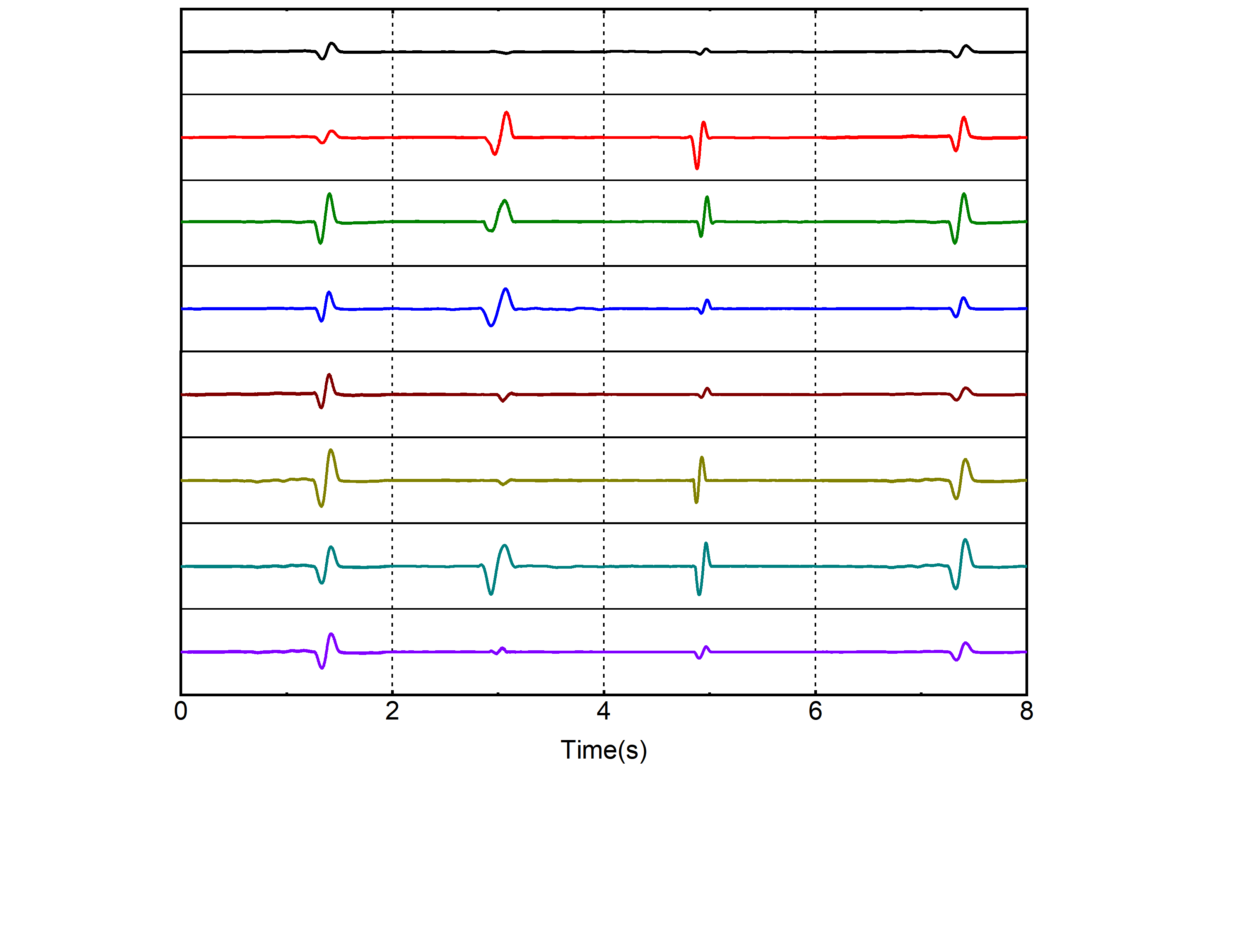


**Line A**

**Line B**

**Line C**

**Line D**

**Line 1**

**Line 2**

**Line 3**

**Line 4**


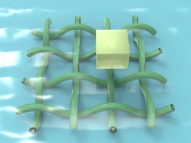

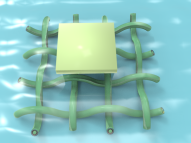

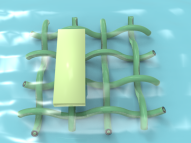

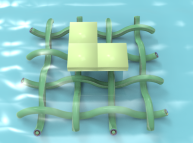

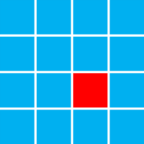

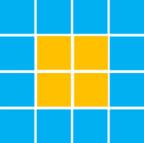

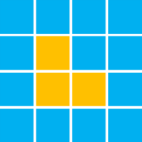

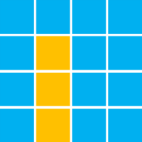


**Figure S15.** Real-time monitoring of important information, such as the momentum, size, and shape of underwater objects.

**Figure note 8.** Whether the complex systematic movement and the physiological signals of the human body can be accurately monitored becomes a crucial criterion for the sensitivity and accuracy evaluation of the CS-TENG sensor. CS-TENG is attached to a specific part of the human body. It generates an electric current when sensing the movement change, which is captured by the data acquisition card and converted into signals using the corresponding data processing software. **Figure S16** shows the response of the self-powered sensing system to the movements of the human body. It can be seen that, when the finger flexes, the range of motion and required force is much less than making a fist, which is also much less than the force produced by the arms bending. When increasing the motion amplitude, the tensile degree of the self-powered sensor increases, the radial shrinkage of the shell structure increases while the distance between the shell and the core structures decreases. An increased external load in the radial direction results in a more sufficient contact between the two triboelectric materials. This is an important factor for distinguishing the different movement states and physiological indexes. In this respect, frequency is another important indicator. The fingers, palms, wrists and elbows perform different functions under the same movement state. When shaking hands, the fingers flexing and wrist shaking are simple movements. However, their frequencies significantly vary. The frequency is used to distinguish different parts, while the simple motion state of each part is analyzed by combining the peak value of the wave. It can also be combined in order to perform the judgment of the complex movement posture. The monitoring of the human body depends on the degree of its fit with the corresponding human body. It can be judged by the waveform of the signal over a period. Moreover, compared with other studies of the literature, the proposed self-powered sensor has the advantage of being able to work underwater for a long time. It can be seen from **Figure S17** that the self-powered sensing system can maintain the same accuracy and sensitivity for the complex underwater motions as it does in the air.


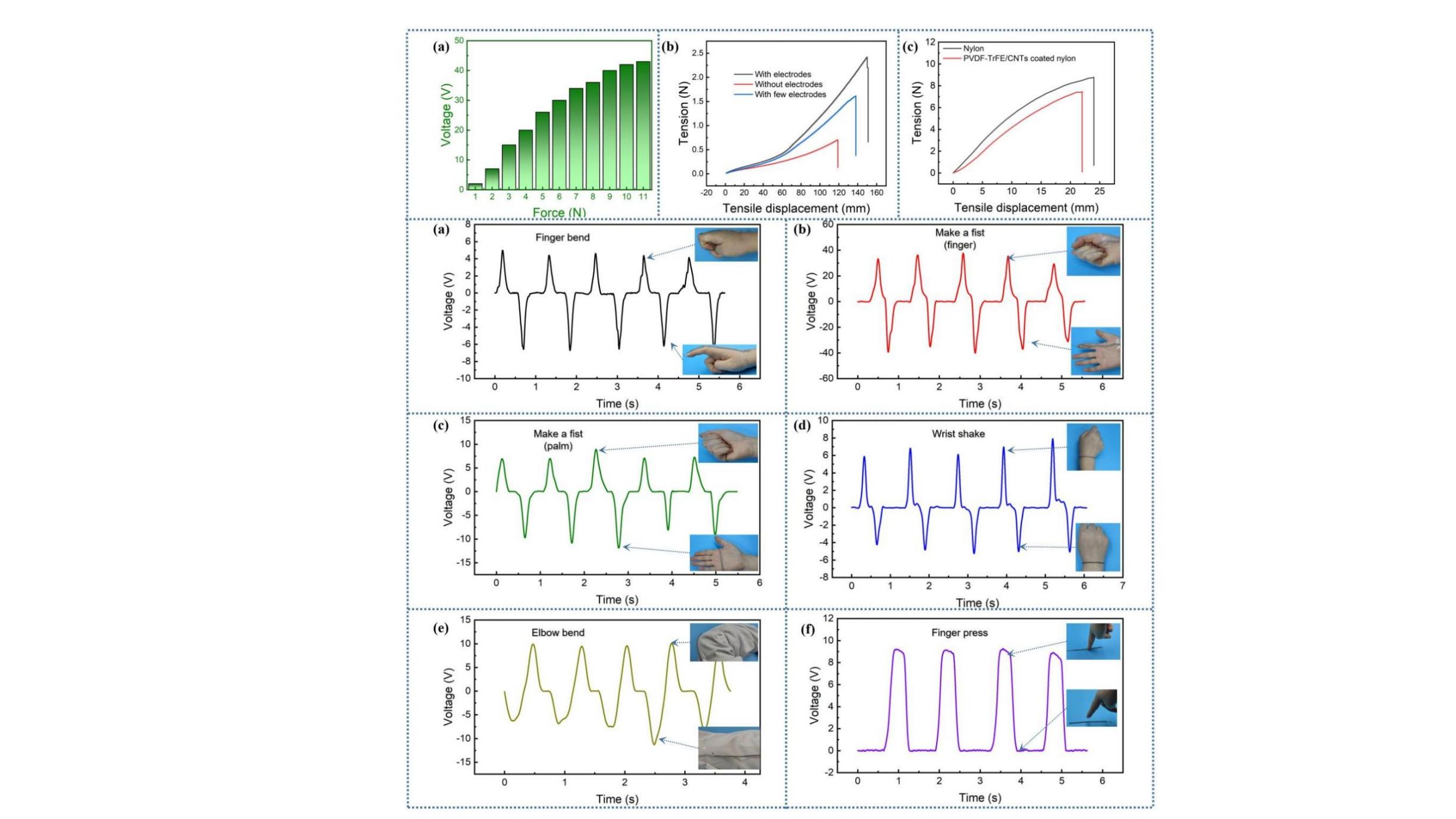


**Figure S16.** Real-time monitoring of human hand and upper limb movement status, including (a) finger bending, (b) changes in the state of fingers when making a fist, (c) changing in the state of palms when making a fist, (d) wrist shaking, (e) elbow bending, and (f) finger pressing.

**
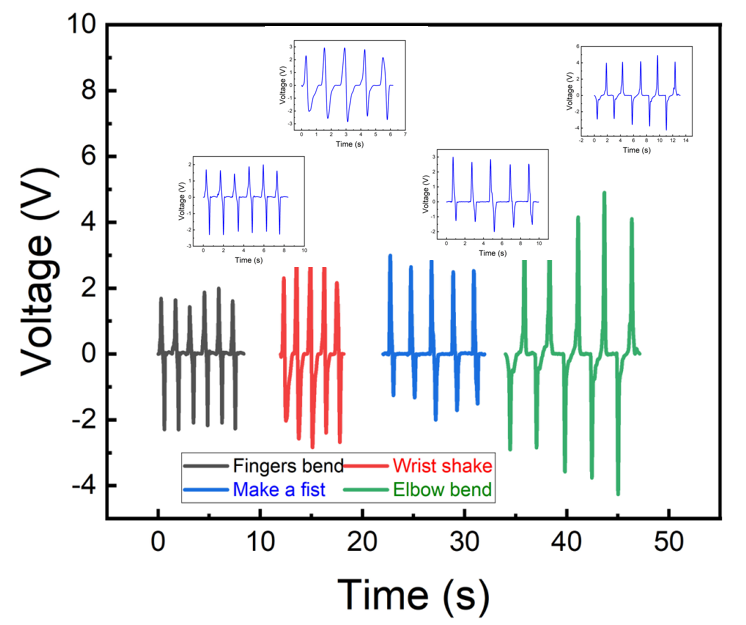
**

**Figure S17.** The self-powered sensor based on CS-TENG is used to monitor the motion state of the underwater human body in real time, including finger bending, wrist shaking, making a fist, and bending elbow.

**References**

[1] C. Ning, K. Dong, R. Cheng, J. Yi, C. Ye, X. Peng, F. Sheng, Y. Jiang, Z. L. Wang, *Advanced Functional Materials* **2020**, 31, 2006679.

[2] W. Gong, C. Y. Hou, Y. B. Guo, J. Zhou, J. K. Mu, Y. G. Li, Q. H. Zhang, H. Z. Wang, *Nano Energy* **2017**, 39, 673.

[3] Y. Cheng, X. Lu, K. H. Chan, R. R. Wang, Z. R. Cao, J. Sun, G. W. Ho, *Nano Energy* **2017**, 41, 511.

[4] Y. Yang, L. Xie, Z. Wen, C. Chen, X. Chen, A. Wei, P. Cheng, X. Xie, X. Sun, *ACS Appl Mater Interfaces* **2018**, 10, 42356.
